# A Rare and Unique Case Report of Lateral Uterine Wall Rupture and Its Review

**DOI:** 10.7759/cureus.38695

**Published:** 2023-05-08

**Authors:** Mukta Agarwal, Smita Singh, Shivangni Sinha

**Affiliations:** 1 Obstetrics and Gynaecology, All India Institute of Medical Sciences, Patna, IND; 2 Obstetrics and Gynecology, All India Institute of Medical Sciences, Patna, IND

**Keywords:** unscarred uterus, misoprostol, scarred uterus, pregnancy, uterine rupture

## Abstract

Rupture of the uterus is a deadly obstetric complication. Its occurrence is uncommon and much less common in the second trimester. Given that the mother and fetus are in danger, it is a catastrophe for both. The incidence has increased in recent years as the cesarean section rate has increased, but in developing nations, multiparity and the inappropriate use of uterotonics are more common. This potentially disastrous event may have a vague initial presentation. Here forth, we present a case with solitary right lateral wall uterine rupture covering the entire length of the uterus, the fetus and placenta enclosed in between the broad ligament leaves, most likely due to injudicious misoprostol use at a private health care center superimposed on multiparity, and a literature review. As far as we know, this is the first instance of an isolated right lateral uterine wall rupture sparing the lower segment and, with the fetus trapped between the broad ligaments simulating abdominal pregnancy.

## Introduction

Uterine rupture is a rare, life-threatening obstetrical condition that describes a rip in the uterine wall involving its whole thickness, including the serosa. The hospital-based incidence of uterine rupture ranges from 1 in 100-500 deliveries in impoverished nations to 1 in 3000-5000 in developed nations [1-3].

The scarred uterus is the most commonly reported etiology of uterine rupture in any trimester, followed by the morbidly attached placenta, grand multipara, and injudicious administration of oxytocin and prostaglandin [4]. In developed countries, the frequency of uterine rupture after a cesarean operation dropped even more, to 1 in 2000 [5], from the global average of 1 in 100 [6]. Occurrence in a non-scarred uterus is extremely rare, with estimates ranging from 1 in 8000 to 1 in 15,000 [7]. Using misoprostol for second-trimester abortion, the risk of rupture is less than 0.35% in the scarred uterus and 0.04% in the unscarred uterus [8]. Multiparity is another predisposing factor for uterine rupture, as repeated pregnancy leads to thinning of the uterine muscle. The maternal mortality rate from uterine rupture is 0.2% in developed countries, while it can reach up to 30% in impoverished countries [9]. Risk of uterine rupture landing into hysterectomy ranges from 14 to 33% [10]. The clinical diagnosis might be difficult since uterine rupture in the first or second trimester of pregnancy is exceedingly rare and varies in presentation and course of events. Nevertheless, uterine rupture should be checked out before considering any other non-gynecological causes in any pregnant woman who comes with severe abdominal discomfort, regardless of gestational age. Other frequent manifestations include vaginal hemorrhage, maternal tachycardia, hypotension, and/or uterine atony, and tenderness in the abdomen [7].

Here, we present a case of a single, right lateral wall uterine rupture that extended along the whole length of the uterus, expelling the fetus and placenta in between the broad ligament leaves. This case was most likely brought on by the improper use of misoprostol at a peripheral healthcare facility, which was then compounded by multiparity. As far as we know, this is the first case of an isolated right lateral uterine wall rupture sparing the lower section and, with the fetus trapped between the broad ligaments, falsely resembling abdominal pregnancy.

## Case presentation

A Gravida 5 Para 4 with Live issue 3 patient with a 23-week gestational period arrived in the emergency room with an abdominal ultrasound report that suggested intrauterine fetal death. Her IUD was verified during a normal prenatal check-up and ultrasound examination four days prior at a private clinic. Misoprostol was used to induce labor at the hospital. She had four doses of misoprostol 400 micrograms, followed by 200, 400, and 200 per vagina over three days, but she did not expel and experienced loose stools and vomiting four to five times each day, so she presented to our emergency labor department.

She had delivered three healthy female children by vagina seven, five, and three years prior. Each of the three deliveries went smoothly. She had undergone a surgical abortion for eight weeks, missed abortion two years prior, and required a three-unit blood transfusion during suction and evacuation to treat anemia.

She was examined and found to have tachycardia, tachypnoea, a normotensive state, a 93% oxygen saturation level, and a fever of 101.7 °F. Clinically she was icteric. According to the abdominal examination, the uterine size was 26 weeks, the uterine shape was maintained, and moderate tenderness was evident. During the vaginal inspection, the area was congested, dry, and heated; the cervical os was also 2 cm dilated; the length was 2 cm; and some fetal parts were palpable.

She was admitted to the intensive care unit (ICU), and all customary tests-including a vaginal swab, blood culture, urine culture, and coagulation profile, were done. Antibiotics meropenem and clindamycin were begun after a clinical diagnosis of sepsis, and the patient was kept on oxygen support and was planned for laparotomy after antibiotics coverage. Her coagulation profile and liver function test results were abnormal. Meanwhile, her blood pressure began to drop, and she was put on inotropes. The immediate decision to do an emergency laparotomy was made. Enough blood product arrangements were made, along with explanations of the requirement for ventilator support and the possibility of an obstetric hysterectomy.

**Figure 1:**
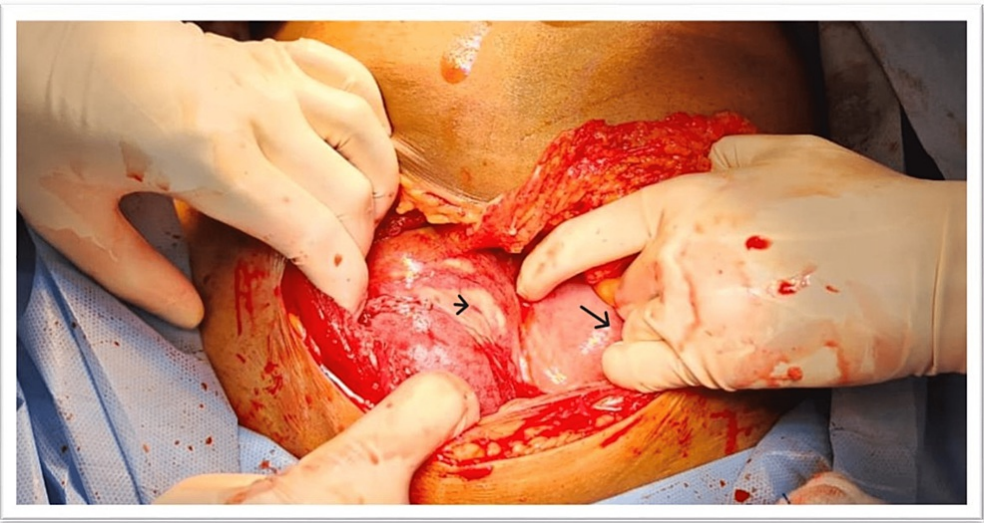
Foetus in a sac distinct from the uterus mimicking abdominal pregnancy in the first instance. Big Arrow: Uterus Small Arrow: Fetus in a different sac

Under general anesthesia, the abdomen was opened by making a suprapubic transverse incision. When the peritoneum was opened, it was discovered that the right side of the uterus had a noticeable lump the same size as the uterus (26 weeks) (Figure 1).

**Figure 2:**
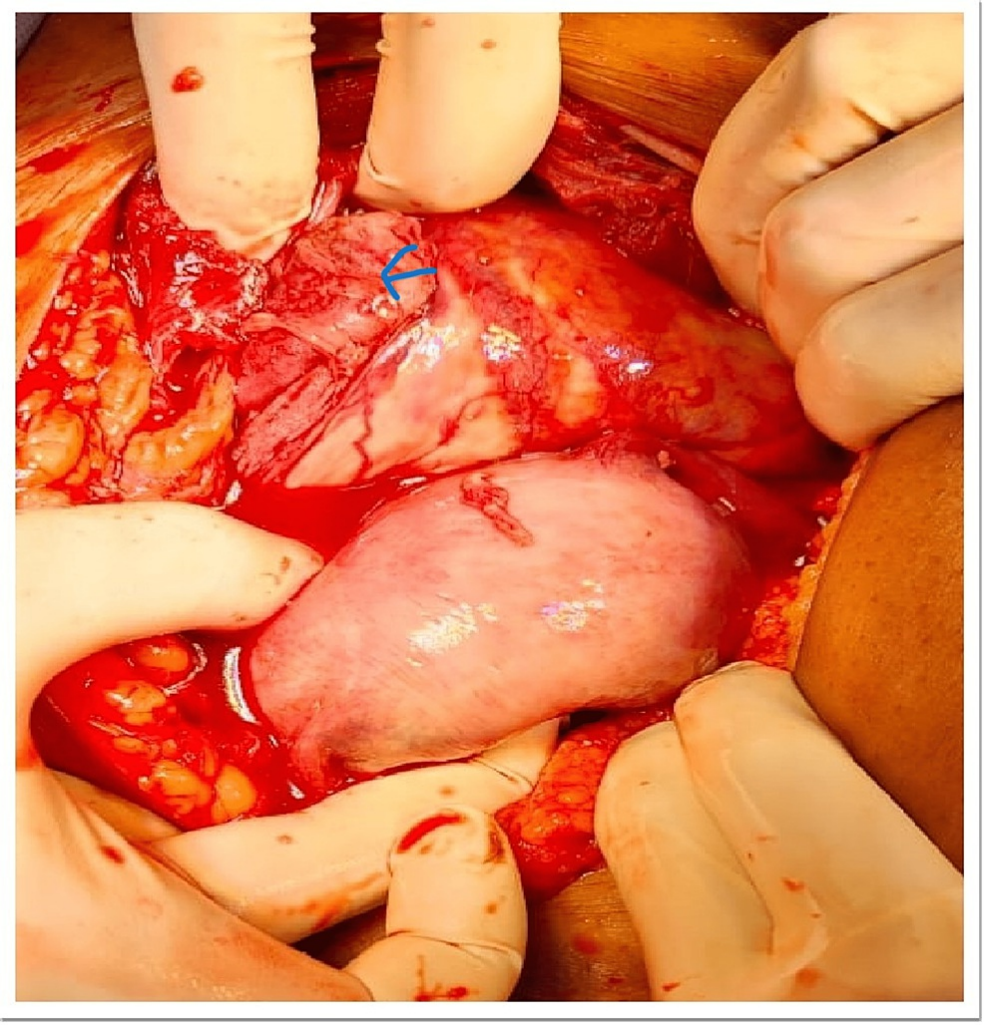
Foetus was seen through a small tear in the anterior leaf. Arrow-Foetus And Placenta Visible in the Sac

**Figure 3:**
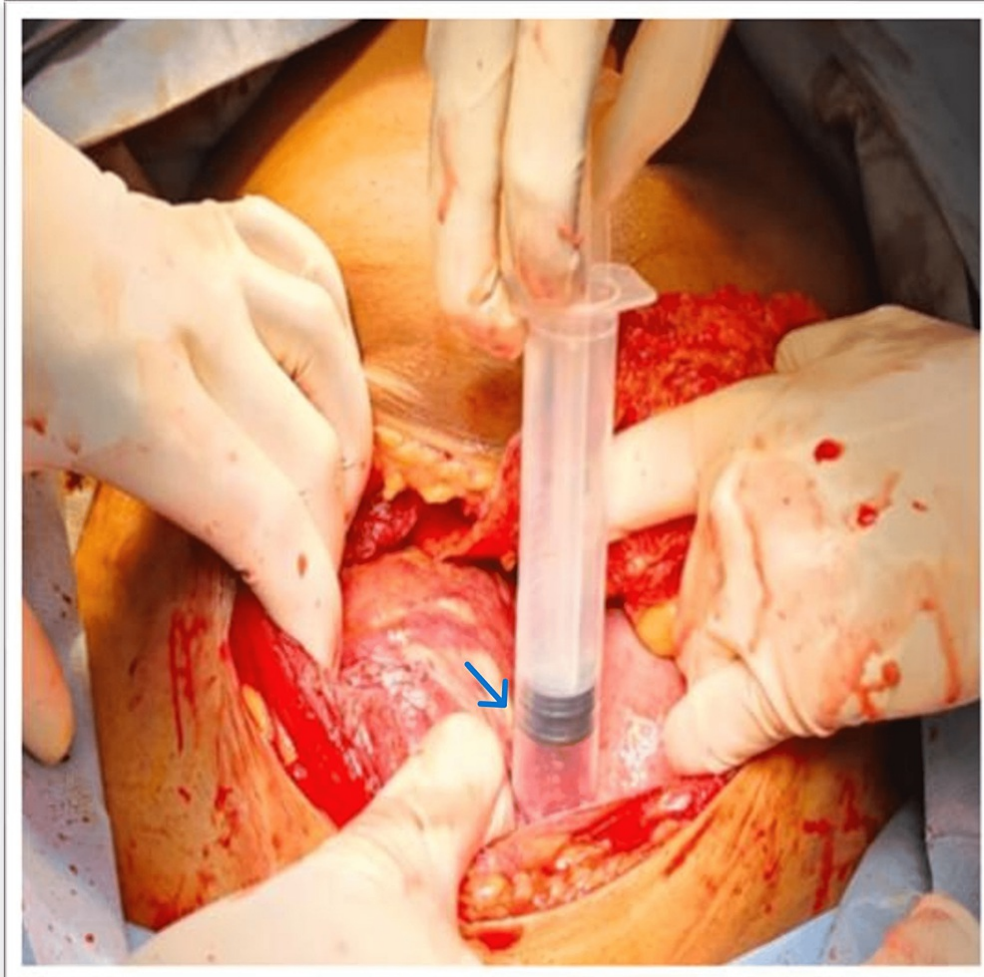
The amniotic sac was filled with pus. An arrow-amniotic sac filled with pus

**Figure 4:**
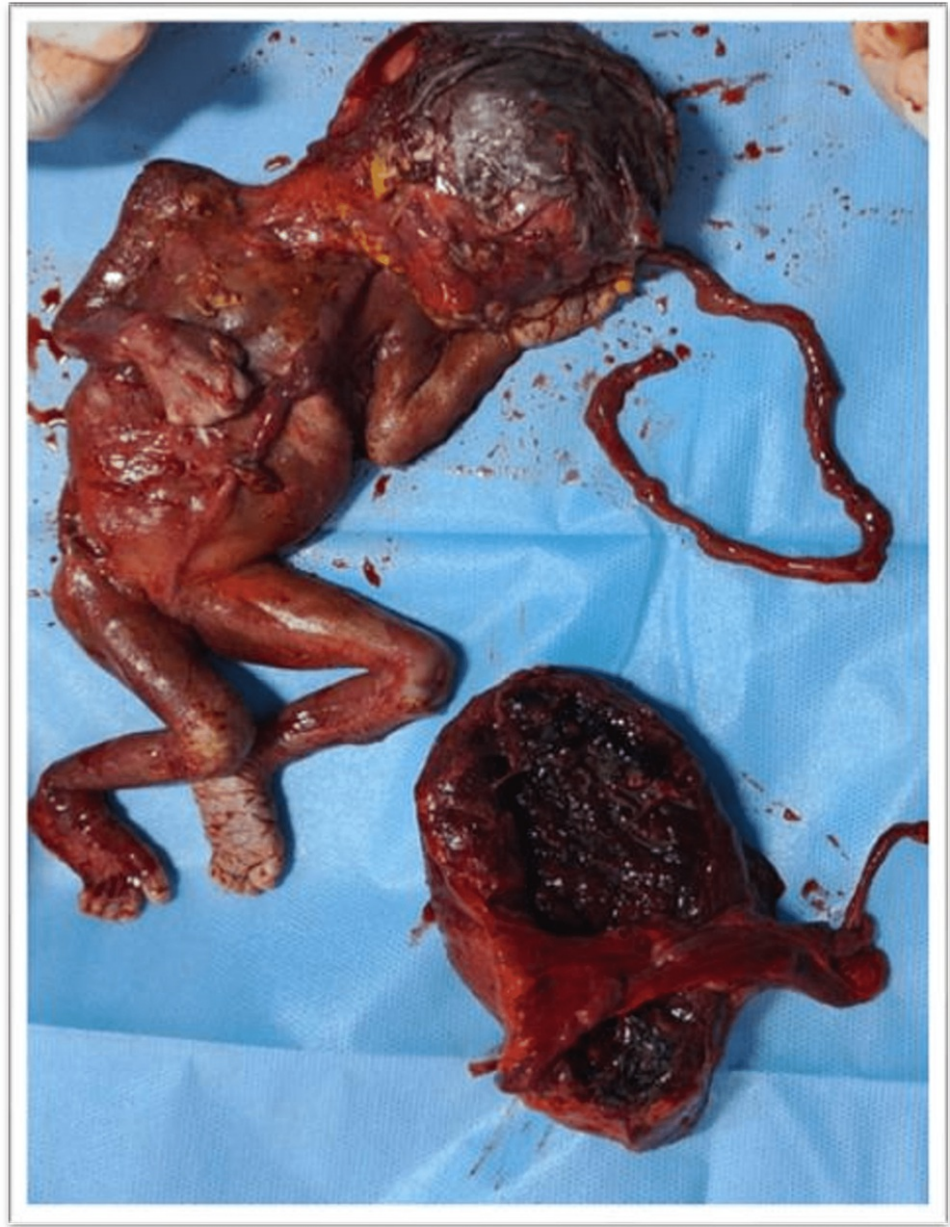
A macerated fetus with the placenta.

**Figure 5:**
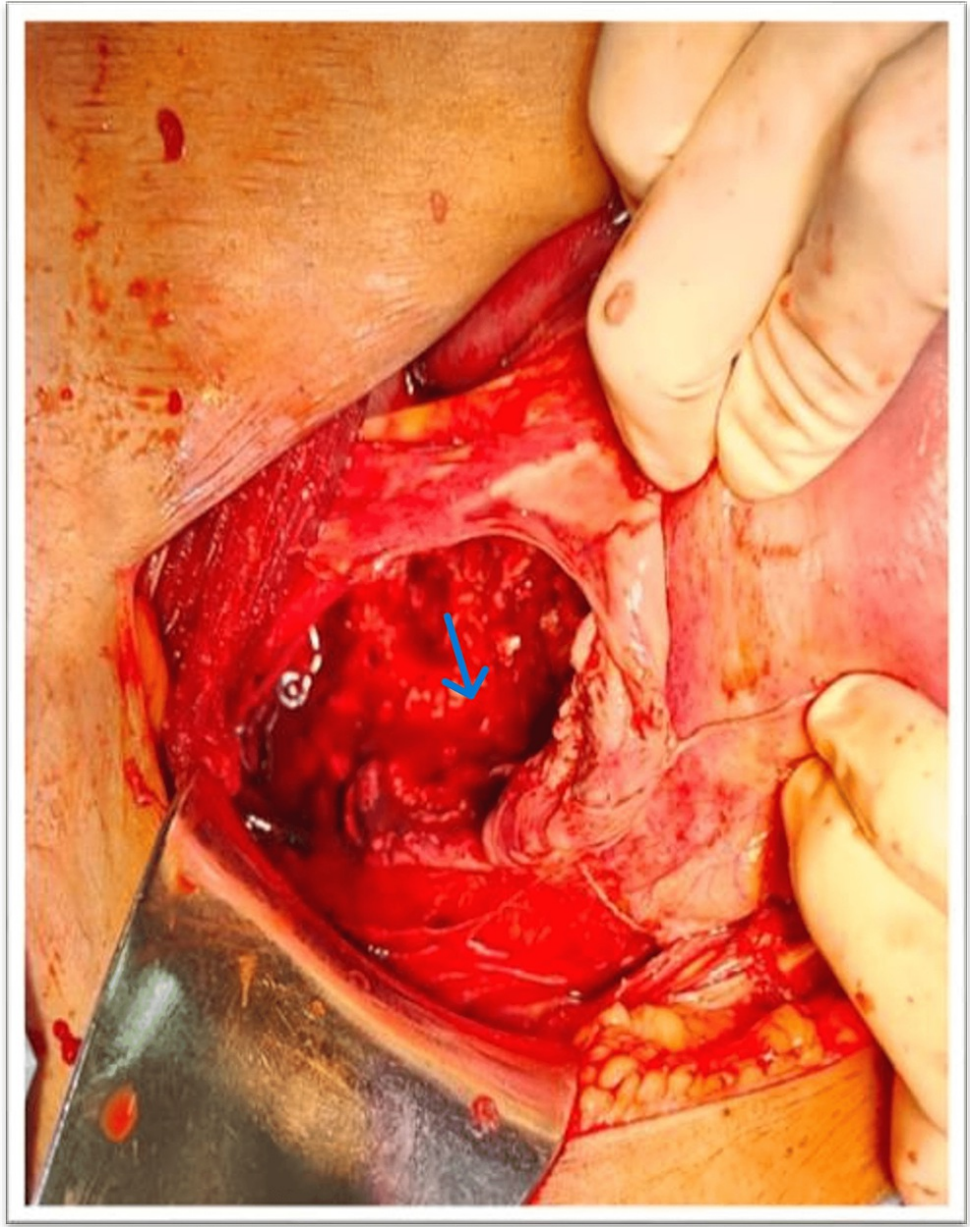
Visible pseudo sac. Arrow-Fetus was in a Pseudo sac Formed Between Two Leaves of Broad Ligament.

At first glance, this suggested abdominal pregnancy, but further handling created a minor rip in the anterior leaf of the broad ligament, which revealed the baby and placenta were resting between the broad ligament's two leaves, simulating abdominal pregnancy (Figure 2). Pus was found in the amniotic sac of the macerated fetus, which was submitted for sensitivity testing and culture (Figure 3). The macerated fetus and the placenta were removed from between the broad ligaments (Figure 4, 5).

**Figure 6:**
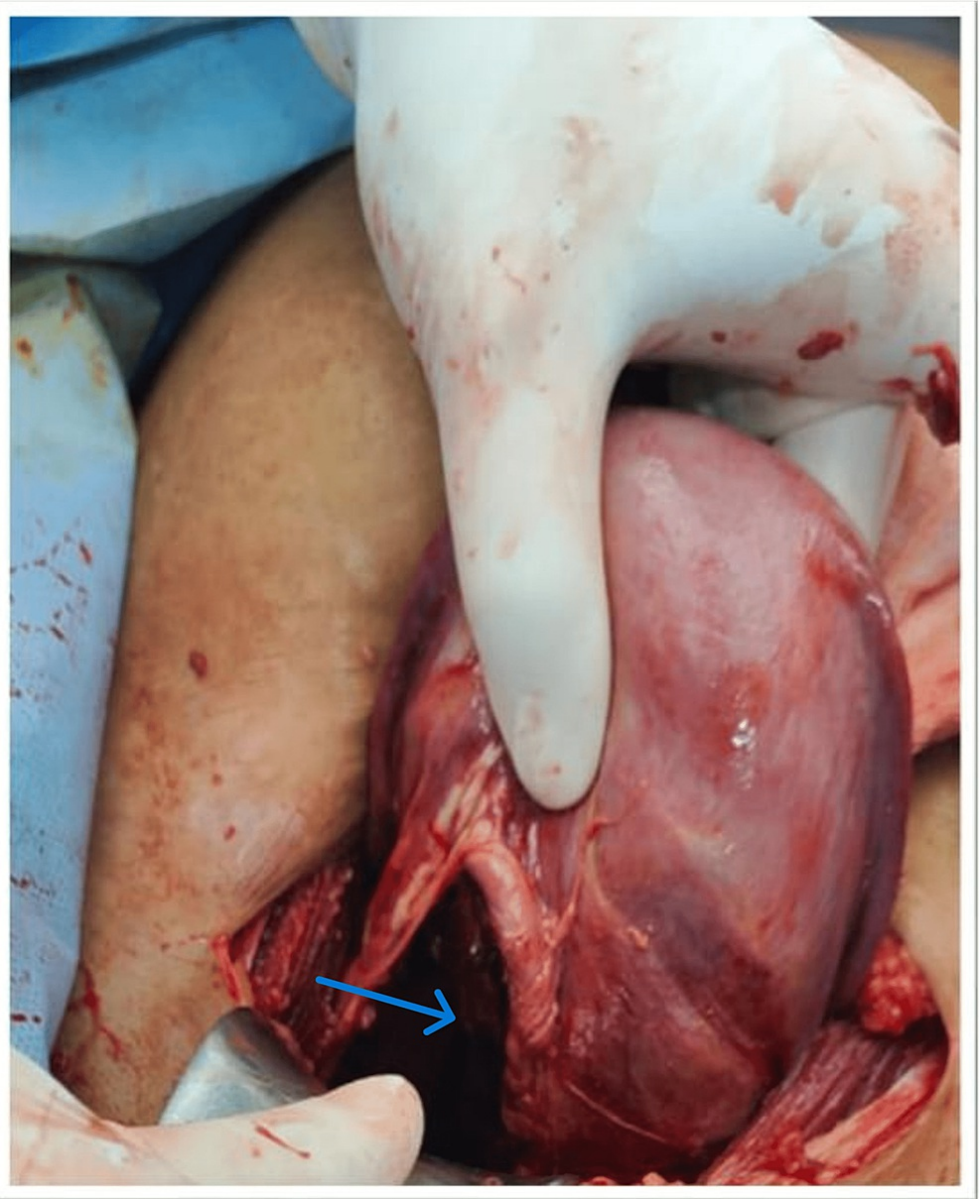
Unrepairable rupture of the right lateral wall Arrow-Rupture Of Right Lateral Uterine Wall

**Figure 7:**
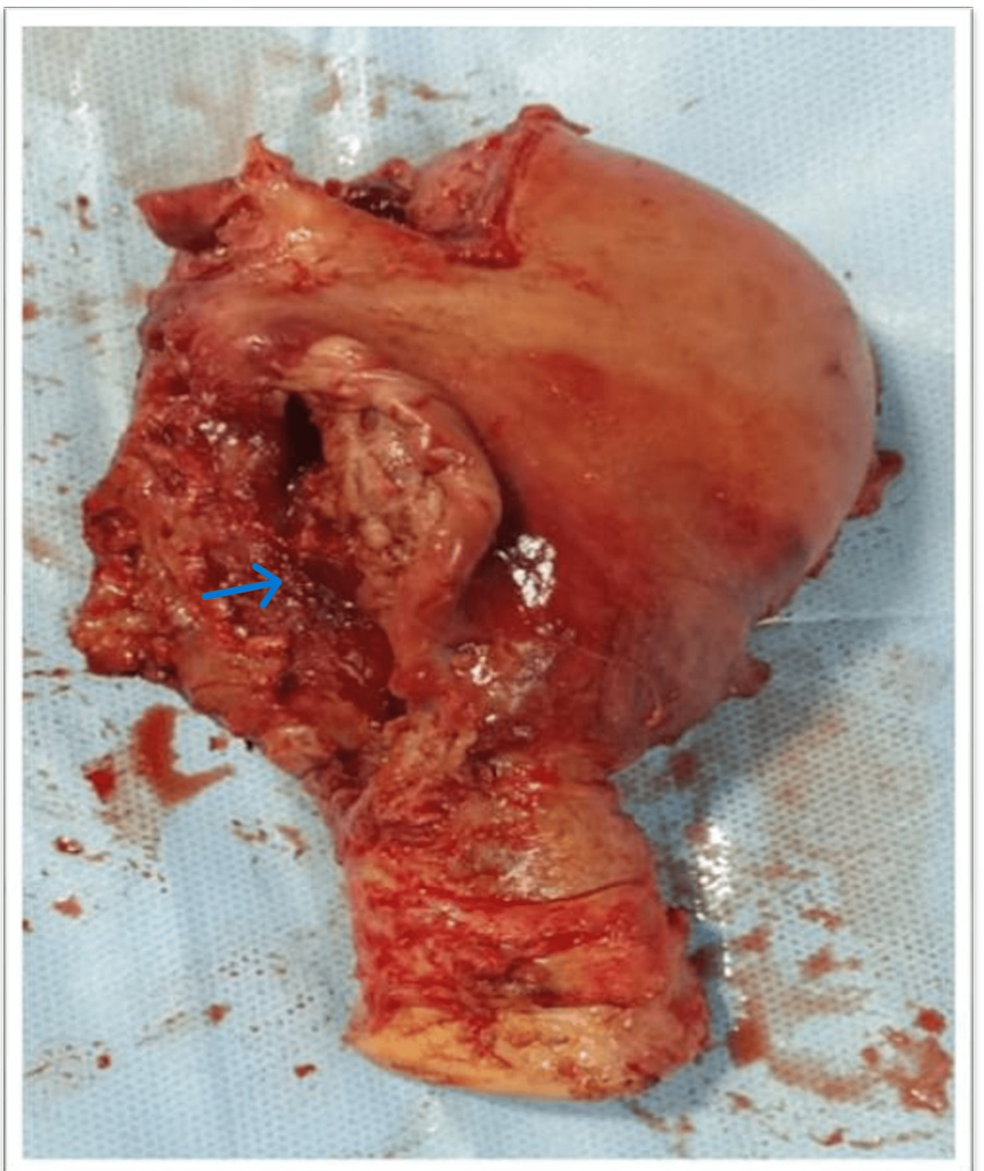
Post Total hysterectomy, visible complete lateral wall tear (arrow)

The whole right lateral wall of the uterus was ripped (Figure 6), which was irreparable, and an obstetric hysterectomy was performed (Figure 7). Two fresh frozen plasma (FFP) infusions were done intraoperatively. The procedure took two hours, and the average amount of blood loss was 1143 ml.

She was kept on ionotropic and oxygen support for 72 hours during the postoperative period. Following surgery, one packed red blood cell (PRBC) unit and two additional FFP were administered. A vaginal swab revealed E. coli, and the antibiotic was switched from meropenem to amikacin based on sensitivity. She was moved from the ICU to the general ward on day four, and on day five, she was discharged in stable condition.

## Discussion

Chances of uterine rupture in second-trimester termination of pregnancy are extremely rare in the unscarred uterus and occur more frequently in developing countries [11]. In a study of uterine ruptures in the Netherlands, the incidence of rupture in unscarred uteruses accounted for only 13 percent of all ruptures, while the incidence in the scarred uterus was 5.1 per 10,000 births [5]. The greatest risk factor for uterine rupture in an unscarred uterus is using oxytocin infusion or prostaglandins, multiparity, prolonged labor, and lack of timely access to emergency obstetrical service [12]. The incidence of ruptured uteri was found to be 0.03% in a large retrospective review of 46 cases over 25 years [13], while According to a population-based study conducted in Norway, the incidence rates of uterine rupture per 10,000 live births were 1.2, 0.9, 1.7, and 6.1 from 1967 to 1977, 1978 to 1988, 1989 to 1999, and 2000 to 2008, respectively [14]. Reported side effects of misoprostol are nausea and vomiting in 2.5% to 34% of patients, fever/hyperthermia in 10% to 30% of patients, and diarrhea in 10% or less of patients [15]. An overview of various recent studies of uterine rupture, its incidence and location, risk factors, rate of hysterectomy, and rate of mortality are mentioned in Table 1.

**Table 1:** Comparison of results of various recent studies of uterine rupture.

Serial no.	Reference	Design & duration	Study population(n)	Incidence	Multiparous	Scarred uterus	Unscarred uterus	Induced labour	Site of rupture	Hysterectomy	Maternal mortality
1.	Zwart et al. [5]	Population-based cohort study, August 2004 - August 2006	210	0.059	203	183	25	Oxytoxin-22, prostaglandin-32	Scar site rupture was MC	17	0
2.	Sinha, Maruti et al. [18]	7-year retrospective analysis	47	1 in 1,633 deliveries (0.061%), 0.318% in scarred uteri, and 0.02% in unscarred uteri	36	33	14	Induced labor-18	MC site-scar rupture	5	0
3.	Veena et al. [19]	Retrospective, July 2008-June 2010	93	0.28%	88 (95%)	71	21	7	-	12	0
4.	Chang et al. [20]	Retrospective, 2008-2018	32	0.084%	26	18	14	Oxytocin-10, OXYTOCIN + prostaglandin-2	Scar site rupture was MC-16	3	0
5.	Vandenberg he et al. [21]	A descriptive multi-country population-based study.	864	0.033.3%	-	0.22%	0.006%	-		87	2
7.	Paprikar S et al. [22]	Retrospective study, July 2018-March 2020	37	0.52%	34	8	29	Oxytocin-6, Prostaglandin-5	Most common (MC) site-Anterior surface of the lower uterine segment (LUS)	3	3
8.	Singh and Shrivastava Study [23]	Prospective cross-sectional study January 2012 to August 2013	40	0.35%		25	15	Oxytocin-21, Misoprostol-3	MC site -LUS	4	1

In 2009, Attarde et al. documented a case of second-trimester abortion with a history of prior LSCS in which the uterus was empty, and the fetus and placental tissue were virtually entirely outside the uterus on ultrasonography. A dead fetus was found during an exploratory laparotomy between two leaves of the broad ligament with a 3 cm uterine wall defect at the location of the prior cesarean scar, which was repaired [16]. Another comparable case was reported by Dhont et al., in which misoprostol was utilized for second-trimester abortion, whereas oxytocin was used in the previously mentioned study [17]. However, our situation is distinct and the first of its kind. Our situation might be summed up as follows: inappropriate use of prostaglandin to terminate a second-trimester dead pregnancy resulted in a quiet rupture of the full length of the right lateral uterine wall, encapsulating the fetus in between the broad ligament leaves like a pseudo sac. Every stage of the diagnosis was flawed since the uterine shape was preserved, and the discomfort was misdiagnosed as typical labor pain.

The common causes of uterine rupture in women who have not experienced scarring are grand multiparity, prolonged or difficult labor, forceps delivery, uterine trauma from prior abortion-related instrumentation, version, and oxytocin stimulation. Other possible causes include iatrogenic uterine perforation during hysteroscopy procedures, a prior salpingectomy, and corneal resection after ectopic pregnancy and myomectomy. In our case, the woman had a previous incident of one uterine curettage for a missed abortion. There is a possibility that she had uterine scarring from an unknown incomplete or complete uterine perforation during curettage, leading to giving way after misoprostol induction. So, the multiparity, misoprostol, and prior uterine curettage history were three distinct risk factors in our case. However, the possibility of uterine rupture before misoprostol induction, which would have resulted in fetal death in this particular case, cannot be ruled out.

## Conclusions

The present case highlights the significance of employing medications like oxytocin and prostaglandins under professional supervision with adequate obstetric practice, competent medical and paramedical staff, and well-equipped healthcare facilities to address complications. Not to mention, establishing proper obstetric practice can help prevent problems like uterine rupture. Patients' survival after uterine rupture is determined by the golden time interval between rupture and intervention, the availability of blood products for transfusion, a good anesthetic faculty, and ICU backup.
